# Adherence to Mediterranean Diet and Implications for Cardiovascular Risk Prevention

**DOI:** 10.3390/nu17121991

**Published:** 2025-06-12

**Authors:** Giulia Frank, Barbara Pala, Paola Gualtieri, Giuliano Tocci, Giada La Placa, Laura Di Renzo

**Affiliations:** 1 PhD School of Applied Medical-Surgical Sciences, Tor Vergata University of Rome, Via Montpellier 1, 00133 Rome, Italy; giulia.frank@ymail.com (G.F.); barbara.pala93@gmail.com (B.P.); laplacagiada@gmail.com (G.L.P.); 2 School of Specialization in Food Science, Tor Vergata University of Rome, Via Montpellier 1, 00133 Rome, Italy; 3 Division of Cardiology, Department of Clinical and Molecular Medicine, University of Rome Sapienza, Sant’Andrea Hospital, Via di Grottarossa 1035/1039, 00189 Rome, Italy; giuliano.tocci@uniroma1.it; 4 Section of Clinical Nutrition and Nutrigenomics, Department of Biomedicine and Prevention, Tor Vergata University of Rome, Via Montpellier 1, 00133 Rome, Italy; laura.di.renzo@uniroma2.it

**Keywords:** Mediterranean diet, cardiovascular disease, 4P medicine, carotid intima-media thickness

## Abstract

**Background/Objectives:**Arterial hypertension, increased carotid intima-media thickness (cIMT), and arterial stiffness (AS) are recognized predictors of cardiovascular disease (CVD). Emerging evidence suggests that vascular remodeling may precede the full development of hypertension. Furthermore, body mass index (BMI), fat mass percentage (FM%), and visceral adipose tissue (VAT), are significant risk factors for cardiovascular events. Conversely, adherence to the Mediterranean diet is associated with reduced cardiovascular risk due to its beneficial effects on lipid metabolism, inflammation, and vascular health. **Methods**: This observational study explored the association between nutritional care and cardiovascular risk in 55 Italian adults (27 women, 28 men) consecutively evaluated at the Section of Clinical Nutrition and Nutrigenomics, University of Rome “Tor Vergata”, in 2024. Nutritional and vascular assessments, including BMI, waist-to-hip ratio (WHR), BIA, DXA, lab tests, blood pressure (BP), pulse wave velocity (PWV), and cIMT, were recorded. Spearman’s rank correlation coefficient was used to evaluate the relationship between vascular and nutritional parameters. Wilcoxon rank sum test; Fisher’s exact test; and Pearson’s Chi-squared test were performed for statistical analysis. Participants were divided into two groups based on cIMT > 0.90 mm and ≤0.90 mm. **Results**: Significant correlations emerged between higher MEDAS scores and BMI (*r* = −0.53, *p* < 0.01), FM% (*r* = −0.49, *p* < 0.01), VAT (*r* = −0.63, *p* < 0.01), and cIMT (*r* = −0.88, *p* < 0.01). Higher WHR and VAT were associated with increased brachial and central BP and PWV. Notable dietary differences were significantly higher between cIMT groups. Total cholesterol/C-HDL, C-LDL/C-HDL, the Atherogenic Index of Plasma, and the HOMA Index differed significantly between groups. Significant differences were also observed in the left ventricular diastolic function (*p* = 0.04), LVM/BSA, and LVM/h^2.7^ in individuals with subclinical atherosclerosis (*p* < 0.05). **Conclusions**: These innovative findings underline the importance of multidisciplinary approaches to prevent CVD and suggest long-term benefits of Mediterranean diet adherence on vascular health.

## 1. Introduction

Cardiovascular disease (CVD) remains a leading cause of morbidity and mortality worldwide, with carotid atherosclerosis being a prevalent condition that significantly contributes to its overall burden [1]. Atherosclerosis plays a pivotal role in the onset of cardiovascular events by promoting plaque formation, vascular inflammation, and arterial narrowing, ultimately leading to ischemic complications such as stroke and myocardial infarction [2]. Among the recognized predictors of CVD [3], high blood pressure (BP) [4], increased carotid intima-media thickness (cIMT) [5], and arterial stiffness (AS) [6] are key indicators of vascular dysfunction and heightened cardiovascular risk. Notably, emerging evidence suggests that vascular stiffening may develop before the full manifestation of hypertension, indicating that early arterial changes could serve as an initial trigger in the progression toward CVD [7].

Additionally, body composition, particularly fat mass, has been increasingly recognized as a critical factor influencing vascular health [8]. Body mass index (BMI) [9], fat mass (FM) [10], and visceral adipose tissue (VAT) [11] are significant risk factors for cardiovascular events. Excess adiposity contributes to systemic inflammation, oxidative stress, and metabolic dysregulation, all of which accelerate atherosclerotic processes and compromise arterial function, further reinforcing the complex interplay between body composition and cardiovascular risk [12].

Diet plays a crucial role in modulating the risk of atherosclerosis by influencing metabolic pathways, systemic inflammation, oxidative stress, and body composition [13]. Specific nutrients contribute to the prevention of chronic degenerative diseases by exerting beneficial effects on lipid metabolism, inflammation, and vascular health [14,15,16]. Unhealthy dietary patterns, characterized by high consumption of saturated fats, refined sugars, and processed foods, promote endothelial dysfunction and lipid accumulation, increasing the risk of atherosclerosis [17]. Conversely, the Mediterranean diet (MedDiet) is associated with improved lipid profiles, reduced inflammation, and enhanced vascular function, thereby lowering the risk of atherosclerosis [18]. The MedDiet is distinguished by a high intake of fresh fruits and vegetables, whole grains, olive oil, and lean protein sources such as fish and poultry, along with moderate consumption of dairy products, particularly yogurt and cheese. It also emphasizes the use of herbs and spices like basil, oregano, and garlic, as well as nutrient-dense nuts and seeds, including almonds and sunflower seeds [19]. Moreover, adherence to the MedDiet has been linked to a significant increase in High-Density Lipoprotein Cholesterol (C-HDL) and upregulation of Apolipoprotein E (APOE) expression, particularly in females, further supporting its cardioprotective effects [20].

Given the role of body composition and the MedDiet in carotid atherosclerosis, this observational study aims to investigate the influence of individual dietary components and specific body composition parameters on different indices of atherosclerosis, with the purpose of identifying potential predictive parameters in the development of the disease.

## 2. Materials and Methods

### 2.1. Study Design

This observational study was conducted between September and December 2024 at the Section of Clinical Nutrition and Nutrigenomics, University of Rome “Tor Vergata”. A total of 55 subjects enrolled in the study gave their consent by reading and signing the informed consent form, in accordance with the Helsinki Declaration of 1975, as revised in 2013. The trial received approval from the Ethics Committee of the Calabria Region Central Area Section (register protocol no. 97, 20 April 2023).

Eligibility criteria included males and females aged 18 to 65 years with a BMI > 19 kg/m^2^. We included individuals with or without arterial hypertension under stable treatment (at least two years), without substantial changes in dietary habit over the past 12 months. Exclusion criteria comprised pregnancy and feeding, BMI exceeding 40 kg/m^2^, being a current smoker, acute illnesses, autoimmune and intestinal disorders, HIV/AIDS, and neoplastic diseases. Participants receiving statin therapy were excluded due to the potential influence on the lipid level and vascular parameters. Patients with a recent diagnosis of arterial hypertension were excluded from the study, as the absence of a stable pharmacological treatment and the lack of assessment of hypertension-mediated organ damage could have influenced the study outcomes, introducing potential confounding factors. Individuals with recent known coronary artery disease or a history of major cardiovascular events—including myocardial infarction or stroke—were also excluded from the study.

All patients underwent an assessment of their pathological history, dietary habits, nutritional status, body composition, and complete cardiovascular evaluation.

### 2.2. Dietary Habits and MedDiet Adherence

The subject’s dietary intake was evaluated using a 24-h recall, while a food frequency questionnaire (FFQ) was administered to determine the weekly consumption patterns of various foods [21]. The FFQ assessed the intake frequency of 36 commonly consumed foods in Italy, along with their portion sizes. Compliance rates were calculated for each food item.

Adherence to the MedDiet was assessed using the validated 14-item Mediterranean Diet Adherence Screener (MEDAS), which assigns a score ranging from 0 to 14 points (Table A1, Appendix A.1) [22]. Based on the MEDAS scores, subjects were classified into the following three adherence categories: low (0–5), medium (6–9), and high (≥10).

### 2.3. Evaluation of Body Composition

Weight, height, and waist circumference were measured following standard protocols [23].

BMI was calculated as body weight (kg) divided by height squared (m^2^) [24]. The waist-to-height ratio (WHR) was determined using a cutoff value of 0.5 [25]. The body adiposity index (BAI) was calculated using the following formula [26]:

BAI = 100 × (hip circumference (m)/(height (m))^1.5^) − 18

Bioelectrical impedance analysis (BIA) was performed using a phase-sensitive system (BIA 101S, Akern/RJL Systems, Florence, Italy) at a frequency of 50 kHz to measure resistance, reactance, impedance, phase angle (PhA), and body cell mass (BCM) following standard procedures [27]. Total body water (TBW) (%) was estimated using the equation proposed by De Lorenzo et al. [28].

Body composition was analyzed at baseline using Dual-Energy X-Ray Absorptiometry (DXA) (Primus, X-ray densitometer; software version 1.2.2, Osteosys Co., Ltd., Gurogu, Seoul, Republic of Korea), following previously described procedures [29]. This assessment included soft tissue evaluation, FM, total body lean mass (LM), bone tissue, total body bone mass (TBB), and VAT [29].

The intramuscular adipose tissue (IMAT) was calculated using the equations proposed by Bauer et al. [30], which are as follows:

For women, Log (IMAT) = −2.21 + (0.12 × FM) − (0.0013 × FM^2^)

For men, Log (IMAT) = −2.05 + (0.12 × FM) − (0.0013 × FM^2^)

### 2.4. Cardiovascular Evaluation

Systolic and diastolic brachial office blood pressure (BP), as well as central (aortic) BP, were assessed non-invasively using a cuff-based oscillometric device (Mobil-O-Graph PWA Monitor, I.E.M. GmbH, Stolberg, Germany), following the European Society of Hypertension guidelines [31]. Central BP was recorded 30 s after brachial BP, and heart rate (HR) was measured simultaneously. Cuff sizes were adapted to participants’ individual arm circumference to ensure accurate readings [31].

Pulse wave velocity (PWV), the gold standard to assess arterial stiffness (AS) in clinical practice, was derived from the time interval between the estimated forward and reflected pressure waves, and has been shown to correlate well with invasive measurements [32].

As AS rises, the velocity of both forward and reflected pressure waves increases, leading to an earlier return of the reflected wave in the central aorta. This earlier arrival enhances central systolic BP during late systole, thereby increasing the Augmentation Index (AI@75) [33]. AI@75 was determined as the ratio between augmentation pressure and pulse pressure (PP), and the values were standardized to a heart rate of 75 beats per minute [34].

All participants underwent a color Doppler ultrasound examination of the supra-aortic trunks (TSA) MyLab™ Seven ultrasound system (Esaote S.p.A., Genoa, Italy). cIMT was measured bilaterally in the common carotid artery at approximately 1 cm proximal to the carotid bifurcation, in accordance with current clinical guidelines [31]. Measurements were taken on the far wall during end-diastole and averaged over multiple cardiac cycles to ensure accuracy.

Individuals underwent transthoracic echocardiography (MyLab™ Seven ultrasound system (Esaote S.p.A., Genoa, Italy)) in the left lateral decubitus position. A single experienced operator performed the exams according to standard guidelines [35]. Measurements including the left atrium diameter, left ventricular (LV) internal dimensions, interventricular septal thickness in diastole (IVSd), posterior wall thickness in diastole (PWTd), and ejection fraction (calculated using the Teichholz method) were assessed. LV geometry was classified into four patterns [36]. Diastolic function was evaluated using Doppler echocardiography and tissue Doppler imaging (TDI), by analyzing the ratio of early (E) to late (A) ventricular filling velocities (E/A), deceleration time, isovolumetric relaxation time (IVRT), and the ratio of early mitral inflow velocity to early diastolic mitral annular velocity (E/e′).

LV mass (LVM) was calculated as follows: LVM = 0.8 × 1.04 × [(IVS + LVEDD + PW)^3^ − LVEDD^3^] + 0.6 g. LVM obtained via echocardiography was normalized to the body surface area (BSA) following the recommendations of the American Society of Echocardiography and the European Association of Cardiovascular Imaging (ASE/EACVI) [37].

In accordance with EACVI guidelines, LVM was also indexed to height raised to the power of 2.7 [38]. Left ventricular hypertrophy (LVH) was defined by LVM/BSA values exceeding 115 g/m^2^ for men and 95 g/m^2^ for women, or by LVM/height^2.7^ values greater than 50 g/m^2.7^ for men and 47 g/m^2.7^ for women.

After an overnight fast (≥8 h), blood samples were collected at baseline and after 8 weeks to assess fasting glucose, insulin, creatinine, and liver enzymes (Glutamic Oxalo-Acetic Transaminase (GOT), Glutamate Pyruvate Transaminase (GPT) and the lipid profile as total cholesterol (C-TOT), C-HDL, Low-Density Lipoprotein Cholesterol (C-LDL)—calculated using the Friedewald equation [39]—and triglycerides (TG).

The Homeostatic Model Assessment for Insulin Resistance (HOMA-IR) was calculated using fasting glucose and insulin levels, according to the following formula: [fasting glucose (mg/dL) × 0.0555 × fasting serum insulin (µIU/mL)]/22 [40].

Moreover, the Atherogenic Index of Plasma (AIP), a reliable marker for assessing the risk of atherosclerosis and CVD, calculated as the logarithm of the ratio between plasma TG and C-HDL levels, was considered [41].

Furthermore, we analyzed Lipoprotein(a) (Lp(a)), which has gained increasing recognition as an independent risk factor for atherosclerotic CVD, due to its pro-inflammatory and pro-thrombotic properties that significantly enhance its atherogenic potential—estimated to be 5 to 6 times greater than that of C-LDL particles on a molar basis. Lp(a) levels vary widely among individuals, ranging from 0.2 to 750 nmol/L (0.1 to 300 mg/dL); however, no universally accepted cutoff exists to define elevated Lp(a), although values above 30 mg/dL have been associated with increased cardiovascular risk [42].

### 2.5. Statistical Analysis

Demographic, pharmacological, anthropometric, and clinical characteristics of patients were reported. No formal sample size calculation was performed, as this was an observational study aimed at exploring associations rather than testing predefined hypotheses.

Descriptive statistics were conducted for the above characteristics. Continuous variables were summarized using the mean and standard deviation (SD), while categorical variables were presented as frequency and percentage. All data were stratified by cIMT ≤ 0.9 mm and cIMT > 0.9 mm, and the appropriate statistical tests were employed to evaluate significant differences between the groups (including Pearson’s Chi-squared test, Kruskal–Wallis rank sum test, and Fisher’s exact test).

Given the non-linear relationships observed between most of the variables, Spearman’s rank correlation was chosen, as it effectively captures monotonic associations regardless of the linearity of the data. Spearman’s correlation coefficient ^®^ ranges from −1 to 1, where absolute values of 0.70 and above indicate a strong correlation, absolute values between 0.40 and 0.70 suggest a moderate correlation, and absolute values below 0.40 represent a weak correlation.

The level of significance was 0.05 and all analyses were explorative and without adjustment of *p*-values. All statistical analyses were performed using R software (version 4.4.2, R Foundation for Statistical Computing, Vienna, Austria).

## 3. Results

The study population included 55 individuals, with a mean age of 52.00 ± 12.00 years and a nearly equal sex distribution (52.7% female). The mean BMI was 27.30 ± 4.70 kg/m^2^. Out of these patients, twenty had arterial hypertension, and among them, three had dyslipidemia without any treatment. There were no other relevant medical conditions in the patients’ medical history, and they were not taking any medications other than anti-hypertensives (Angiotensin II Receptor Blockers and/or Calcium Channel Blockers). All of these demographic, pharmacological, and anthropometric parameters are summarized in Table 1.

The average FM% was 28.00 ± 9.00%, with a corresponding Fat-Free Mass (FFM) of 55.00 ± 13.00 kg. Handgrip strength averaged 35.00 ± 11.00 kg, and the PhA was 5.89 ± 0.88°. The VAT-to-subcutaneous fat (SAT) ratio (VAT/SAT) was 0.34 ± 0.17.

Metabolic parameters, including fasting glucose and cardiovascular indices, were within standard ranges. However, it has to be underlined that the total cholesterol levels were at the upper limit according to the latest ESC cardiovascular prevention [43], with a mean total cholesterol of 213 ± 43.00 mg/dL and LDL cholesterol of 131.00 ± 33.00 mg/dL.

The BP and AS parameters were within normal ranges according to the latest ESC Guidelines. AS, measured by PWV, averaged 7.87 ± 1.02 m/s, and Systolic and Diastolic BP measured 130.00 ± 16.00 mmHg and 84.00 ± 10.00 mmHg, respectively [31].

Finally, the mean cIMT was within the normal range, averaging 0.80 mm. All of these parameters are reported in Table A2 (Appendix A).

According to the latest 2023 guidelines, asymptomatic intimal-medial thickening is considered a sign of organ damage if the cIMT measured by ultrasound exceeds 0.90 mm [31]. Therefore, by dividing the study population into two groups, we observed that 32 individuals had normal cIMT (≤0.90 mm) and 23 individuals had increased cIMT > 0.90 mm.

Key cardiovascular risk markers, including C-TOT/C-HDL, C-LDL/C-HDL and Atherogenic Index of Plasma (AIP), were significantly different between groups (*p* = 0.03; 0.04; 0.02, respectively). There were no statistical differences between the groups’ C-LDL and TG levels. The HOMA Index was statistically significantly higher in the cIMT > 0.90 mm group compared to the other group.

**Figure 1:**
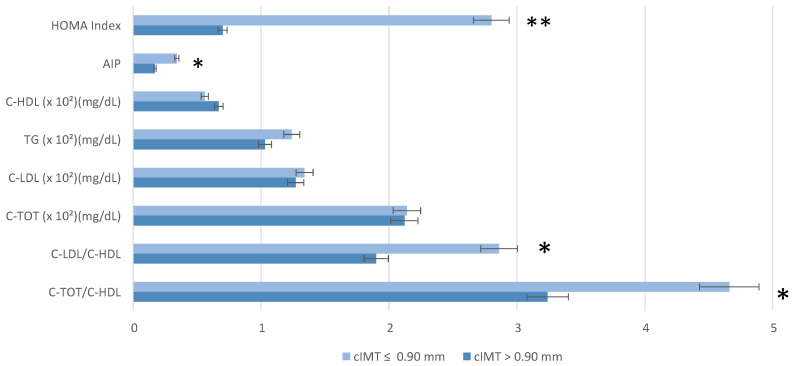
Cardiometabolic risk parameters (lipid profile and (Homeostasis Model Assessment index) stratified by carotid intima-media thickness (cIMT). Wilcoxon rank sum test; Fisher’s exact test; and Pearson’s Chi-squared test were performed for statistical analysis. *p* < 0.05 *; *p* = 0.01 **. Abbreviation: AIP, Atherogenic Index of Plasma; C-HDL, Cholesterol—high-density lipoporotein; C-LDL, Cholesterol—low-density lipoporotein; C-TOT, Total Cholesterol; C-TOT/C-HDL, Total Cholesterol/high-density lipoporotein; C-LDL/C-HDL, Cholesterol—low-density lipoporotein/Cholesterol—high-density lipoporotein.

A comparison of cardiometabolic risk parameters between the groups is represented in Figure 1.

**Figure 2:**
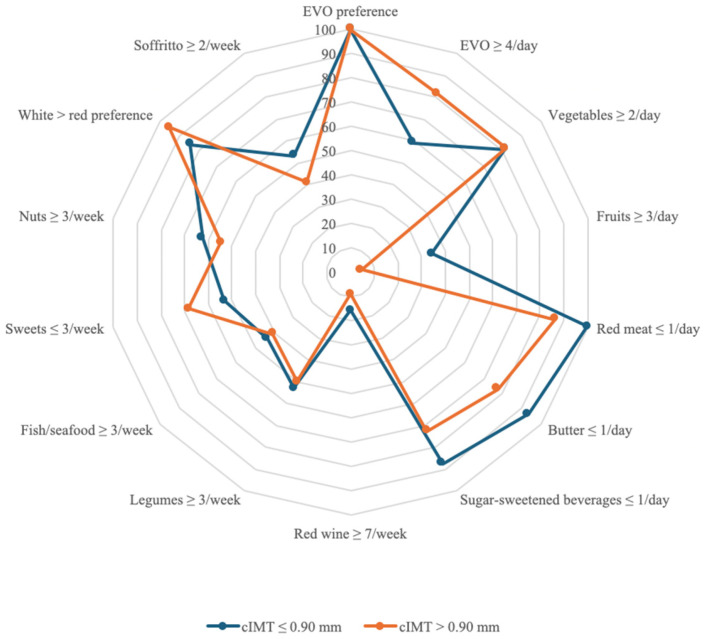
Answers and scoring of the MEDAS. The “YES” answer to the MEDAS items is compared between the sample with cIMT ≤ 0.90 mm and the group with cIMT > 0.90 mm. The radar chart shows for each MEDAS score item the affirmative responses of the sample with cIMT ≤ 0.90 mm compared to the group with cIMT > 0.90 mm with separate axes starting from the center of the draft (0% “YES” responses) and at the end of the last circle (100% “YES” responses). Values are in percentages. Abbreviations: EVO, extra virgin olive oil; cIMT, carotid intima-media thickness.

Additionally, the MEDAS score was significantly higher in individuals with lower cIMT (*p* = 0.03), supporting the association between MedDiet adherence and lower subclinical atherosclerosis risk [44]. The MEDAS responses between the groups are reported in Table 2 and Figure 2.

Notable dietary differences included fresh cheese consumption, which was significantly higher in the lower cIMT group (*p* = 0.03 monthly). Regarding red meat consumption, a statistically significant difference was found (*p* = 0.03). Similarly, the intake of sausage and processed meat products was significantly different between groups (*p* = 0.03). Lastly, extra virgin olive oil (EVO) consumption was also significantly different (*p* < 0.01), with higher adherence to EVO intake in the cIMT ≤ 0.90 mm group.

**Figure 3:**
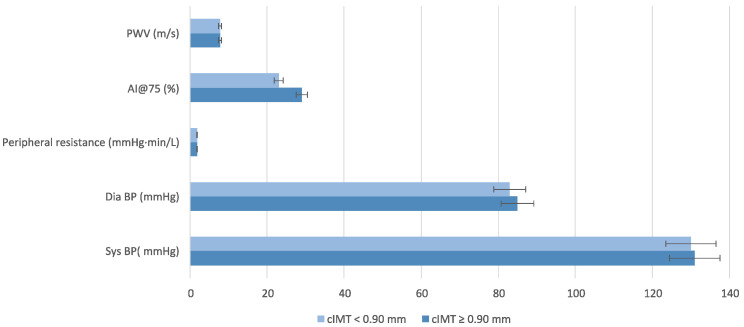
Blood Pressure Parameters stratified by carotid intima-media thickness (cIMT). Wilcoxon rank sum test; Fisher’s exact test; Pearson’s Chi-squared test was performed for statistical analysis. Abbreviation: AI@75, Augmentation Index; cIMT, intima-media thickness; PWV, pulse wave velocity.

Findings regarding BP and AS highlight trends rather than statistically significant differences (Figure 3).

**Figure 4:**
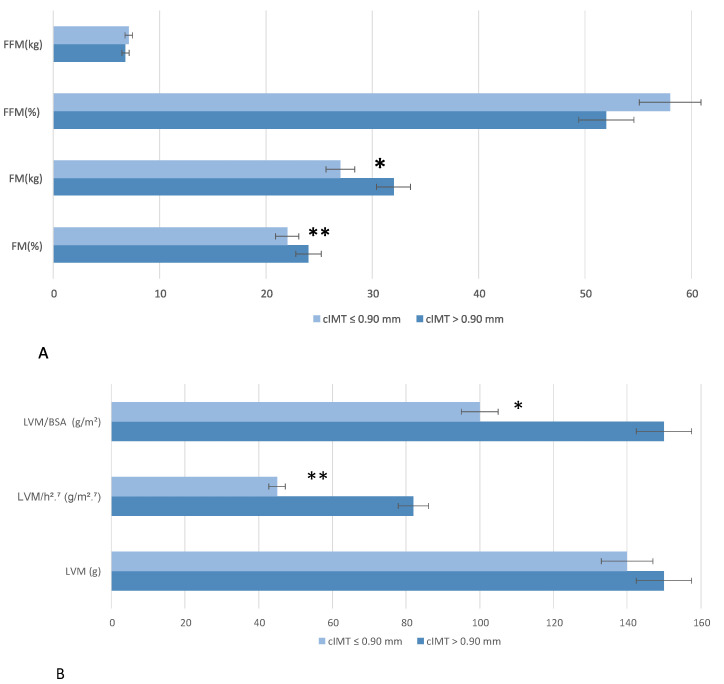
Bioimpedentiometric (**A**) and ecocardiographic (**B**) parameters stratified by carotid intima-media thickness (cIMT). Wilcoxon rank sum test; Fisher’s exact test; Pearson’s Chi-squared test was performed for statistical analysis. *p* < 0.5 *; *p* < 0.01 **. Abbreviation: BSA, body surface area; FFM, Fat-Free Mass; FM, fat mass; LVM, left ventricular mass.

There were no significant differences between the two groups in terms of FM, FM%, FFM, or FFM% (Figure 4A). No significant differences were found in the absolute LVM between groups (*p* = 0.32); however, when indexed to body surface area (LVM/BSA), a significant difference was observed (*p* = 0.04), with lower values in the cIMT ≤ 0.90 mm group compared to the cIMT > 0.90 mm group. Additionally, when normalized by height^2.7^ (LV MASS/h^2.7^), the cMT > 0.90 mm group had significantly higher values (*p* < 0.01), (Figure 4B).

Finally, significant differences were observed in LV diastolic function (*p* = 0.04) (66.7% of individuals had I grade diastolic disfunction in the cIMT > 0.9 mm and 35.5% of individuals had I grade diastolic disfunction in the cIMT ≤ 0.9 mm).

Spearman’s rank correlation coefficient was used to evaluate the relationship between variables. First, a strong inverse correlation was observed between the MEDAS score and carotid cIMT (*r* = −0.88, *p* < 0.001).

Moreover, a significant negative correlation was observed between weekly legume consumption and Lp(a) levels (*r* = −0.83, *p* < 0.001). Additionally, weekly legume consumption was positively correlated with FT3 (free-Triiodothyronine) levels (*r* = 0.72, *p* < 0.01). A negative correlation was found between MEDAS score and BMI (*r* = −0.53, *p* < 0.001), as well as VAT (*r* = −0.63, *p* < 0.001). Higher MEDAS scores were significantly associated with lower BAI (*r* = −0.43, *p* = 0.001), lower FM% (*r* = −0.49, *p* < 0.001), and lower FM (kg) (*r* = −0.50, *p* < 0.001). Conversely, higher adherence to the MEDAS score correlated positively with FFM% (*r* = 0.44, *p* < 0.001).

A strong positive correlation was observed between BCM and FFM (*r* = 0.92, *p* < 0.001). Similarly, IMAT and FM were significantly correlated (*r* = 0.92, *p* < 0.001), indicating that higher IMAT is associated with increased FM levels. Conversely, FM% was strongly and inversely correlated with FFM% (*r* = −0.92, *p* < 0.001) and BCM% (*r* = −0.89, *p* < 0.001). Regarding muscle strength, FFM (kg) was positively correlated with maximal handgrip strength (*r* = 0.74, *p* < 0.001).

Furthermore, VAT showed a positive correlation with central diastolic BP (*r* = 0.30, *p* = 0.03), systolic BP (Sys) (*r* = 0.36, *p* <0.01), and central systolic BP (c-Sys) (*r* = 0.37, *p* < 0.01). Moreover, VAT was strongly correlated with PWV (*r* = 0.63, *p* < 0.001).

Moreover, WHR was positively correlated with PWV (*r* = 0.33, *p* = 0.01), central diastolic (c-dia) BP (*r* = 0.62, *p* < 0.001), systolic BP (*r* = 0.58, *p* < 0.001), and c-Sys BP (*r* = 0.48, *p* < 0.01).

**Figure 5:**
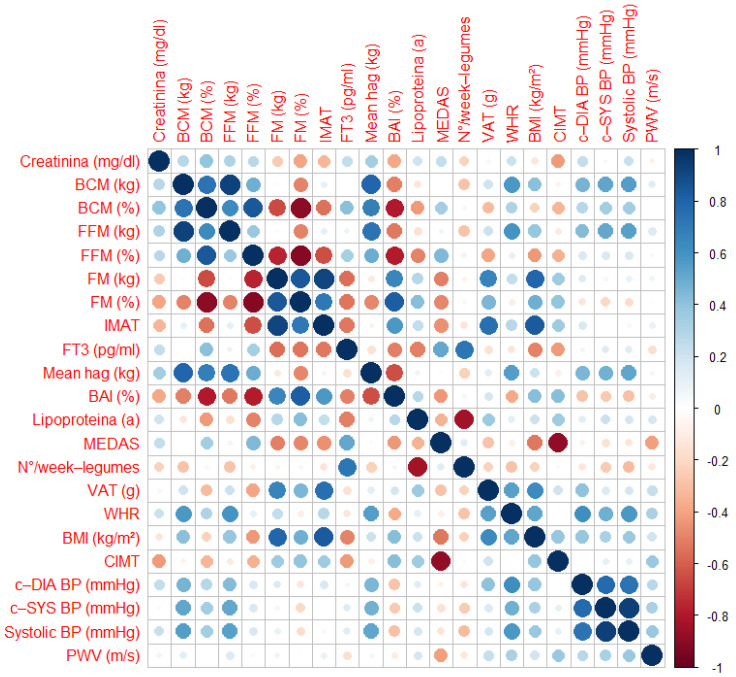
Matrix correlations. Abbreviations: BAI, Body Adiposity Index; BCM, Body Cell Mass; BMI, Body Mass Index; c-DIA BP, Central Diastolic Blood Pressure; c-SYS BP, Central Systolic Blood Pressure; CIMT, Carotid Intima-Media Thickness; FFM, Fat-Free Mass; FM, Fat Mass; FT3, Triiodothyronine; IMAT, Intramuscular Adipose Tissue; MEDAS, Mediterranean Diet Adherence Screener; PWV, Pulse Wave Velocity; Systolic BP, Systolic Blood Pressure; VAT, Visceral Adipose Tissue; WHR, Waist-to-Height ratio.

All correlations are summarized in Figure 5.

## 4. Discussion

The results of this study provide further evidence on the protective role of adherence to the MedDiet in the prevention of CVD. Numerous epidemiological and clinical studies have shown that high adherence to the MedDiet is associated with a reduction in the incidence of cardiovascular events, including myocardial infarction and stroke [45,46,47]. This protective effect is attributable to multiple mechanisms, including reducing oxidative stress, modulating inflammation, and improving lipid profiles [48,49]. Indeed, previous and recent studies deeply investigated the connection between the Mediterranean diet and vascular aging [50,51].

In particular, our study’s novelty lies in its focus on a well-characterized Italian sample with a long-standing, stable adherence to the Mediterranean lifestyle. This was objectively confirmed through both the MEDAS and CCF scores. Such dietary stability may offer a more accurate snapshot of the cardiovascular system’s response to long-term lifestyle exposure. We believe that this context enhances the clinical relevance of our findings by suggesting that simple dietary screening tools may help to identify individuals who would benefit from targeted cardiovascular assessments and personalized preventive strategies. Specifically, according to our knowledge, our data showed a significant inverse association between the MEDAS score and cIMT (*r* = −0.88, *p* < 0.001), an indicator of subclinical atherosclerosis and an early marker of vascular damage [31]. This result suggests that greater adherence to the MedDiet is associated with decreased progression of subclinical atherosclerosis, strengthening the evidence that a diet rich in foods typical of the Mediterranean model may exert a protective role on vascular health, potentially mediated by reducing systemic inflammatory status and improving lipid metabolism [18,52,53].

While more extensive dietary instruments may capture a broader range of food items, shorter tools, such as ours, have demonstrated adequate validity when tailored to the cultural and dietary context of the population studied. Therefore, we believe that the combination of the culturally adapted FFQ and the MEDAS score provides a robust and pragmatic estimate of dietary habits in our cohort [21,22].

Analysis of dietary habits showed significant differences between the groups divided according to cIMT. Consumption of fresh cheese was significantly higher in the group with cIMT ≤ 0.90 mm than in the group with cIMT > 0.90 mm (*p* = 0.03), suggesting a possible role of the type of dairy products consumed in modulating cardiovascular risk [54]. Similarly, red meat consumption showed a statistically significant difference between groups (*p* = 0.03), with higher intake in subjects with cIMT > 0.90 mm, consistent with previous studies that have associated high red meat intake with increased atherosclerotic risk [55,56]. Consumption of sausages and processed meat products was also significantly different between groups (*p* = 0.04), indicating the potential negative contribution of these foods on vascular health [57,58]. Moreover, EVO consumption was significantly higher in the group with cIMT ≤ 0.90 mm (*p* < 0.01), supporting the hypothesis that regular intake of EVO is associated with beneficial effects on the cardiovascular system due to its antioxidant and anti-inflammatory properties [59,60,61]. Furthermore, a significant negative correlation was observed between weekly legume consumption and Lp (a) levels (*r* = −0.83, *p* < 0.001), suggesting a potential hypolipidemic effect associated with legume intake [62]. Considering that Lp(a) is a known cardiovascular risk factor for which there are currently no specific pharmacological therapies, these data indicate that targeted dietary strategies could be an effective option to reduce atherosclerotic risk through independent and adjuvant mechanisms to conventional pharmacotherapy [63].

Our results are consistent with a study on peritoneal dialysis patients, which demonstrated that soy isoflavones—bioactive compounds found in legumes—significantly reduced Lp(a) levels, supporting the potential lipid-lowering effects of legume components [64].

To the best of our knowledge, our study reports, for the first time, that the mean MEDAS score achieved by patients with cIMT ≤ 0.90 mm was significantly higher than the group with cIMT > 0.90 mm, confirming the protective role of the MedDiet [44].

Remarkably, FM% was significantly higher in the group with cIMT > 0.90 mm (32%) than in the group with cIMT ≤ 0.90 mm (27%), suggesting a potential correlation between the increased adipose component and progressive deterioration of vascular function [65]. This association could reflect the pro-atherogenic role of adipose tissue, which, through production of pro-inflammatory cytokines and insulin resistance, contributes to AS and the progression of atherosclerosis [66,67,68]. Although this difference did not reach statistical significance, the observed trend in the slightly increase of FM (Kg) could indicate a role of the adipose compartment in impaired vascular health. Previous studies have shown that fat accumulation, particularly at the visceral level, is associated with increased cardiovascular risk, mediated by increased chronic inflammatory status and alterations in lipid and glucose metabolism [69,70].

Our data show that increased VAT is associated with higher BP values, both brachial Sys (*r* = 0.36, *p* < 0.01) and, especially, central sys-diastolic (*r* = 0.37, *p* < 0.01, *r* = 0.30, *p* = 0. 03; respectively), considering the latter to be an important early marker of vascular damage [31]. This report emphasizes how fat accumulation in the abdominal region can negatively affect vascular function, contributing to increased BP, consistent with previous studies [71]. In addition, we were the first to demonstrate a strong correlation between VAT and PWV (*r* = 0.63, *p* < 0.001), a key indicator of AS. These findings suggest that VAT excess not only increases arterial pressure, but also affects arterial elasticity, leading to increased stiffness and reduced vascular adaptability [72]. While previous studies have highlighted the association between VAT accumulation and elevated BP, data directly linking VAT to arterial stiffness have been lacking [73]. This underscores both the novelty and the clinical relevance of our findings. Similarly, WHR, a simple but effective anthropometric measure, was found to be positively correlated with PWV (*r* = 0.33, *p* = 0.01), brachial Sys (*r* = 0.58, *p* < 0.001), and central systolic–diastolic (*r* = 0.48, *p* < 0.01; *r* = 0.62, *p* < 0.001, respectively) BP values.

It is important to emphasize that composite indices of inflammation and cardiovascular risk (C-TOT/C-HDL, C-LDC-L/HDL, and AIP) were found to be more sensitive and predictive than isolated values of C-LDL and TG that were not significant. This underscores the importance of a more integrated assessment of the individual cardiovascular risk profile, which considers not only traditional lipid parameters, but also combined markers such as C-TOT/C-HDL, C-LDC-L/HDL, and AIP [74,75]. Particularly, the most recent 2019 guidelines on dyslipidaemia emphasize that lipid-lowering therapy should not be initiated based solely on absolute cholesterol values, but rather on a comprehensive evaluation of the individual’s overall cardiovascular risk [76].

In addition, the HOMA Index, indicative of insulin resistance, was significantly higher in the group with cIMT > 0.90 mm, highlighting a possible metabolic component associated with increased mid-intimal thickness [77]. Indeed, improving insulin sensitivity before the onset of type 2 diabetes mellitus may help to prevent or slow down the development and progression of atherosclerosis [78].

Regarding cardiac remodeling, LVM is indicated for body surface area (LVM/BSA, *p* = 0.04), and, especially, when indexing LVM values to an elevated height at 2.7 (LVM/h^2.7^, *p* < 0.01), a statistically significant difference was found between the two groups. Our data, in line with data in the literature [79], suggest an initial process of cardiac remodeling in subjects with signs of subclinical atherosclerosis, even in the absence of obvious changes in absolute LVM. Moreover, findings regarding BP and AS highlight trends rather than statistically significant differences, suggesting that while individuals with higher cIMT may have slightly elevated BP and altered vascular parameters, the differences require further investigation with a larger sample size.

Overall, the data obtained support the hypothesis that targeted nutritional interventions based on the adoption of the MedDiet may be an effective strategy for primary prevention of CVD.

Adherence to such a dietary regimen not only improves the lipid and metabolic profile, but also appears to modulate early parameters of subclinical vascular and cardiac damage.

### Limitations

However, this study has some limitations, such as the observational nature and the small sample size (*n* = 55). Therefore, despite the emerging evidence, these limitations do not allow for a definitive causal relationship between MedDiet adherence and cardiovascular risk reduction to be established. Thus, prospective studies on larger cohorts will be needed to confirm the predictive value of the MedDiet in preventing atherosclerosis and reducing long-term cardiovascular risk.

In addition, about one-third of the included subjects (20 of 55) had hypertension under pharmacological treatment. Although all were at the target pressor according to guidelines, the presence of hypertension, even if controlled, may have influenced some vascular and cardiac markers (e.g., cIMT, PWV, LVM), introducing a potential bias in subclinical cardiovascular risk assessment.

It should also be considered that anti-hypertensive therapy might have protective effects on vascular and cardiac remodeling, partially masking any differences between the groups.

Furthermore, the discovery of initial signs of organ damage, despite the anti-hypertensive treatment, suggests the usefulness of a broader and earlier risk assessment based on integrated parameters and a multidisciplinary approach. It also reinforces the idea that early and integrated indicators are critical to intercept residual risk, even in already treated patients.

Since moderate changes in lifestyle, including diet, may influence cardiovascular risk over time, we selected participants who reported stable dietary patterns, not only over the previous year, but also at the time of evaluation. Nevertheless, the potential influence of unreported or subtle changes in lifestyle cannot be entirely ruled out, which remains a limitation of the study.

Moreover, physical activity, although not assessed in our study, may act synergistically with a healthy diet to enhance cardiovascular benefits. Conversely, in individuals with poor dietary habits, regular physical activity might partially compensate for some of the associated metabolic risks. The absence of physical activity data therefore limits our ability to fully interpret the interaction between lifestyle factors and cardiovascular outcomes.

Longitudinal studies will be needed to confirm these findings and assess the evolution of cardiovascular damage over time.

## 5. Conclusions

This study confirms the protective role of adherence to the MedDiet in the prevention of CVD through multiple mechanisms. Higher MedDiet adherence, reflected by a higher MEDAS score, was significantly associated with lower cIMT, suggesting a beneficial effect in preventing subclinical atherosclerosis. Regular consumption of key MedDiet foods, such as extra virgin olive oil, legumes, and fish, was linked to better body composition and reductions in cardiovascular risk factors, partiularly Lp(a).

Additionally, higher VAT accumulation was associated with increased AS and BP, reinforcing the importance of fat distribution and lipid metabolism in CVD progression. The analysis of cardiac function revealed early signs of LV remodeling in individuals with increased cIMT.

These findings highlight the importance of early monitoring of cardiovascular and metabolic parameters and support the implementation of personalized nutritional strategies based on the MedDiet. Further large-scale studies are needed to confirm these associations and explore the potential of nutrigenomic approaches in optimizing CVD prevention. Overall, promoting the MedDiet, combined with comprehensive cardiovascular risk assessment, may represent an effective strategy to reduce the burden of CVD.

## Figures and Tables

**Table 1:** Demographic, pharmacological, and anthropometric parameters.

	*n* Sample = 55
Males	29.00 (52.70)
Females	26.00 (47.30)
Age	52.00± 12.00
BMI (kg/m^2^)	27.30 ± 4.70
Neck circumference (cm)	36.00 ± 3.81
Waist circumference (cm)	87.00 ± 12.00
Abdomen circumference (cm)	95.00 ± 10.00
Hip circumference (cm)	101.00 ± 8.00
WHR	0.86 ± 0.10
Left arm circumference (cm)	31.10 ± 3.60
Left middle thigh circumference (cm)	53.10 ± 5.40
Thigh left root circumference (cm)	60.00 ± 9.00
Wrist circumference (cm)	18.13 ± 8.78
Bicipital Fold (mm)	11.00 ± 5.90
Tricipital Fold (mm)	18.00 ± 8.00
Subscapular fold (mm)	20.00 ± 8.00
Suprailiac fold (mm)	13.51 ± 6.20
Dislipidemia (%)	5.45
Anti-hypertensive Drugs (%)	36.36

Values are expressed as the mean and standard deviation (M ± SD) for continuous variables, or as numbers and percentages (*n* (%)) for categorical variables. Abbreviations: BMI, body mass index; WHR, waist hip ratio.

**Table 2:** MEDAS questionnaire.

	cIMT ≤ 0.90 mm (*n* = 32)	cIMT > 0.90 mm (*n* = 23)
EVO preference	32 (100.00)	22 (100.00)
EVO ≥ 4/day	19 (59.40)	18 (81.80)
Vegetables ≥ 2/day	26 (81.25)	18 (81.80)
Fruits ≥ 3/day	11 (34.40)	1 (4.50)
Red meat ≤ 1/day	32 (100.00)	19 (86.40)
Butter ≤ 1/day	30 (93.75)	17 (77.30)
Sugar-sweetened beverages ≤ 1/day	28 (87.50)	16 (72.70)
Red wine ≥ 7/week	5 (15.60)	2 (9.10)
Legumes ≥ 3/week	17 (53.10)	11 (50.00)
Fish/seafood ≥ 3/week	14 (43.75)	9 (40.90)
Sweets ≤ 3/week	17 (53.10)	15 (68.20)
Nuts ≥ 3/week	20 (62.50)	12 (54.50)
White > red preference	27 (84.40)	21 (95.40)
Soffritto ≥ 2/week	17 (53.10)	9 (40.90)
MEDAS Score	9.25 ± 2.01	8.63 ± 1.74
Low Adherence	1	0
Medium Adherence	15	15
High Adherence	16	7

Data are expressed as number and percentage in parenthesis (*n* (%)) for categorical variables. Vegetables daily serving: 1 medium portion = 200 g. Fruit daily serving: 1 serving = 100–150 g portion. Red meat/hamburgers/other meat daily serving: 1 medium portion = 100–150 g. Butter, margarine, or cream daily serving: 1 medium portion = 12 g. Sweet or sugar sweetened carbonated beverages daily serving: 1 medium portion = 200 mL. Red wine daily serving: 1 medium portion = 125 mL. Legumes weekly serving: 1 portion = 150 g. Fish daily serving: 1 medium portion = 100–150 g. Seafood daily serving: 1 medium portion = 200 g. Nuts weekly serving: 1 portion of dairy product = 30 g. Abbreviations: EVO, extra virgin olive oil; cIMT, carotid intima-media thickness; MEDAS, Mediterranean Diet Adherence Screener.

## Data Availability

This published article includes all of the data generated or analyzed during this study.

## Abbreviations

The following abbreviations are used in this manuscript:

AI@75	Augmentation index normalized to a heart rate of 75 beats per minute
AIP	Atherogenic Index of Plasma
APOE	Apolipoprotein E
AS	Arterial Stiffness
BAI	Body Adiposity Index
BCM	Body Cell Mass
BIA	Bioelectrical Impedance Analysis
BMI	Body Mass Index
BP	Blood Pressure
BSA	Body Surface Area
c-Dia BP	Central Diastolic Blood Pressure
C-HDL	High-Density Lipoprotein Cholesterol
cIMT	Carotid Intima-Media Thickness
C-LDL	Low-Density Lipoprotein Cholesterol
c-Sys BP	Central Systolic Blood Pressure
C-TOT	Total Cholesterol
CVD	Cardiovascular Diseases
DXA	Dual-Energy X-Ray Absorptiometry
EVO	Extra Virgin Olive Oil
FFM	Fat-Free Mass
FFQ	Food Frequency Questionnaire
FM	Fat Mass
FT3	Free triiodiotironine
GOT	Glutamic Oxalo-Acetic Transaminase
GPT	Glutamate Pyruvate Transaminase
HDL	High-Density Lipoprotein
HR	Heart Rate
HOMA-IR	Homeostatic Model Assessment for Insulin Resistance
IMAT	Intramuscular Adipose Tissue
IVRT	Isovolumic Relaxation Time
IVSd	Inter-Ventricular Septum Thickness
LA	Left Atrial
LDL	Low-Density Lipoprotein
LM	Lean Mass
Lp(a)	Lipoprotein (a)
LV	Left Ventricular
LVH	Left Ventricle Hypertrophy
LVM	Left Ventricle Mass
LVM/BSA	Left Ventricle Mass/Body surface area
LVM/h^2.7^	Left Ventricle Mass/height^2.7^
MEDAS	Mediterranean Diet Adherence Screener
MedDiet	Mediterranean Diet
PhA	Phase Angle
PWTd	Posterior Wall Thickness
PWV	Pulse Wave Velocity
SAT	Subcutaneous Adipose Tissue
SD	Standard Deviation
Sys	Systolic Blood Pressure
TBB	Total Body Bone Mass
TBW	Total Body Water
TDI	Tissue Doppler Imaging
TSA	Supra-Aortic Trunks
VAT	Visceral Adipose Tissue
WHR	Waist-To-Hip Ratio

## Appendix A

### Appendix A.1

The MEDAS questionnaire is reported in Table A1.

**Table A1:** MEDAS.

Questions	Yes = 1/No = 0
1. Is olive oil the main culinary fat used?	
2. Are four tablespoons of olive oil used each day?	
3. Are two servings (of 200 g each) of vegetables eaten each day?	
4. Are three servings of fruit (of 80 g each) eaten each day?	
5. Is <1 serving (100–150 g) of red meat/hamburgers/other meat products eaten each day?	
6. Was <1 serving (12 g) of butter, margarine, or cream eaten each day?	
7. Is <1 serving (330 mL) of sweet or sugar-sweetened carbonated beverages consumed each day?	
8. Are three glasses (of 125 mL) of wine consumed each week?	
9. Are three servings (of 150 g) of legumes consumed each week?	
10. Are three servings of fish (100–150 g) or seafood (200 g) eaten each week?	
11. Is <3 servings of commercial sweets/pastries eaten each week?	
12. Is one serving (of 30 g) of nuts consumed each week?	
13. Is chicken, turkey, or rabbit routinely eaten instead of veal, pork, hamburger, or sausage?	
14. Are pasta, vegetable, or rice dishes flavored with garlic, tomato, leek, or onion eaten ≥twice a week?	

### Appendix A.2

In Table A2 all of the BIA, DXA, blood test, and cardiovascular parameters of the sample are reported.

**Table A2:** Sample Parameters.

	*n* Sample = 55
FM% (Siri)	28.00 ± 9.00
BAI (%)	28.60 ± 5.50
Mean handgrip max strenght (Kg)	35.00 ± 11.00
PhA (°)	5.89 ± 0.88
Z (ohm)	532.00 ± 92.00
FM (kg)	23.00 ± 9.00
FM (%)	29.00 ± 9.00
FFM (kg)	55.00 ± 13.00
FFM (%)	70.00 ± 10.00
BCM (kg)	30.00 ± 8.00
BCM (%)	39.00 ± 7.00
BCMI (kg/m^2^)	10.50 ± 2.18
Total Fat (g)	22.17 ± 9.77
Total Lean (g)	51.56 ± 11.41
FM %	28.00 ± 11.00
IMAT	0.91 ± 0.45
ASMMI	8.03 ± 1.24
VAT/SAT	0.34 ± 0.17
HGB (g/dL)	13.79 ± 1.18
HTC (%)	41.55 ± 2.69
Glycemia (mg/dL)	93.00 ± 13.00
Insulin (uUI/mL)	8.42 ± 2.12
C-TOT (mg/dL)	213.00 ± 43.00
C-HDL (mg/dL)	61.00 ± 22.00
C-LDL (mg/dL)	131.00 ± 33.00
TG (mg/dL)	115.00 ± 52.00
GOT (U/lt)	28.00 ± 19.00
GPT (U/lt)	26.00 ± 14.00
Azotemia (mg/dL)	27.00 ± 10.00
Creatinine (mg/dL)	0.83 ± 0.12
Uric Acid (mg/dL)	4.97 ± 1.23
Lipoprotein(a)	43.00 ± 38.00
TSH microUI/mL	2.17 ± 1.00
ft3/ft4	2.71 ± 1.26
PTL	308.17 ± 363.55
Lymphocytes	2.08 ± 464.00
Neutrophilis	3.32 ± 905.00
Homa-Index	1.63 ± 4.62
TyG	8.47 ± 0.50
tg/hdl	2.40 ± 1.97
C-TOT/C-HDL	4.03 ± 1.96
C-LDL/C-HDL	2.47 ± 1.58
AIP	0.26 ± 0.32
PLR	148.00 ± 150.00
NLR	1.66 ± 0.51
Systolic BP (mmHg)	130.00 ± 16.00
Diastolic BP(mmHg)	84.00 ± 11.00
PAM	105.00 ± 12.00
Pulse Pressure (mmHg)	47.00 ± 11.00
Heart Rate (1/min)	68.00 ± 10.00
cSis (mmHg)	120.00 ± 21.00
cDia (mmHg)	85.00 ± 11.00
Peripheral Resistance (Dyn × s/cm^5^)	1.81 ± 236.00
AI@75	26.00 ± 11.00
PWV (m/s)	7.87 ± 1.02
cIMT	0.83 ± 0.16

Abbreviations: ASMMI, Appendicular Skeletal Muscle Mass Index; BAI, Body Adiposity Index; BCM, Body Cell Mass; BCMI, Body Cell Mass Index; cIMT, common carotid artery intima-media thickness; FFM, Fat-Free Mass; FM, Fat Mass; FT3/FT4, Triiodothyronine free/thyroxine free, HGB, Hemoglobin; HTC (%): Hematocrit; Glycemia (mg/dL): Blood Glucose; Insulin (uUI/mL): Insulin; C-TOT (mg/dL): Total Cholesterol; C-HDL (mg/dL): High-Density Lipoprotein Cholesterol; C-LDL (mg/dL): Low-Density Lipoprotein Cholesterol; TG (mg/dL): Triglycerides; GOT (U/L): Glutamate Oxaloacetate Transaminase (AST); GPT (U/L): Glutamate Pyruvate Transaminase (ALT); Azotemia (mg/dL): Blood Urea Nitrogen; Creatinine (mg/dL): Serum Creatinine; Uric Acid (mg/dL): Uric Acid; TSH micro UI/mL: Thyroid Stimulating Hormone; fT3/fT4: Free Triiodothyronine / Free Thyroxine; PTL: Platelet Count; Lymphocytes: Lymphocyte Count; Neutrophils: Neutrophil Count; HOMA-Index: Homeostatic Model Assessment of Insulin Resistance; TyG: Triglyceride-Glucose Index; tg/hdl: Triglycerides to HDL Cholesterol Ratio; Ctot/hdl: Total Cholesterol to HDL Cholesterol Ratio; LDL/HDL: LDL Cholesterol to HDL Cholesterol Ratio; AIP: Atherogenic Index of Plasma; PLR: Platelet-to-Lymphocyte Ratio; NLR: Neutrophil-to-Lymphocyte Ratio; Systolic BP (mmHg): Systolic Blood Pressure; Diastolic BP (mmHg): Diastolic Blood Pressure; PAM: Mean Arterial Pressure; Pulse Pressure (mmHg): Pulse Pressure; Heart Rate (1/min): Heart Rate; cSis (mmHg): Central Systolic Blood Pressure; cDia (mmHg): Central Diastolic Blood Pressure; Peripheral Resistance (Dyn·s/cm⁵): Systemic Vascular Resistance; AI@75: Augmentation Index PWV (m/s): Pulse Wave Velocity; IMAT, Intramuscular Adipose Tissue; PhA, Phase Angle; VAT/SAT, Visceral Adipose Tissue-Subcutaneous Adipose Tissue; Z, Impedance.
